# Cardiovascular Risk-Factors in the Eastern Iranian Population: Are We Approaching 25×25 Target?

**Published:** 2016-02-24

**Authors:** Masoud Siadat, Toba Kazemi, Morteza Hajihossen

**Affiliations:** ^a^ Atherosclerosis and Coronary Artery Research Center, Birjand University of Medical Sciences, Birjand, Iran; ^b^ South Khorasan Health Insurance, Birjand, Iran

## Dear Editor-in-Chief

Non-communicable diseases (NCDs) account for 43% of the global burden of diseases where low- and middle-income countries are involved in 79% of morbidity and 85% of the burden of these diseases^1^. In 2000, WHO proposed its Global Strategy for the Prevention and Control of NCDs. WHO disseminated its global status report on NCDs in 2010 and issued its Political Declaration on NCDs in order to make prevention and control policies for NCDs on the global scale. Then the prevention project was brought to the General Assembly of the United Nations, and decision was made and approved by presidents of several countries including Iran to demand nations to plan for reduction of NCDs burden. It was decided that nations should follow policies whereby 25% of non-communicable premature deaths of individuals between 30 and 70 yr of age would be reduced by 2025; it was called 25×25 target.

WHO maintains that 25×25 target cannot be achieved unless NCDs risk-factors reduced to a certain level. More specifically, the 25×25 target requires a reduction in hypertension about 25%, salt/Na intake nearly 30%, tobacco use 30%, physical inactivity 10%, harmful use of alcohol 10%, diabetes and obesity 0%, and fat intake 15%. The question is whether Iran can manage to reach the target by the year 2025 so that there will be a 25% decreased rate of premature morbidity from NCDs. To answer this question, we enquired and compared a number of NCDs’ risk-factors in Southern Khorassan across some time periods.

We examined three groups of Birjand population, eastern Iran, consisting of Imam Foundation relief seekers in 2008 (mean age=39 ±16.8 yr), nurses in 2011 (mean age=40.6 ±7.2 yr) and a randomly selected sample of people in 2014 (mean age=36.7 ±0.45 yr) ^2,3^. Since 2008, none of the risk factors were declined (Table 1). On the contrary, obesity has reached from a rate of 10.7% to 18.8%. Dyslipidemia, especially low HDL, has been on the rise in a significant way. All these suggest that we cannot achieve the Target.

**Table 1:** Comparison of cardiac risk factors in 3 groups in Southern Khorassan-East of Iran

**Population**	**Year**	**Hypertensio** **n (%)**	**Diabetes** **(%)**	**Obesity** **(%)**	**Smoking** **(%)**	**High LDL** **(%)**	**Low HDL** **(%)**	**Dyslipidemia (%)**
Low socioeconomic population	2008	13.1	6.3	10.7	9.8	43.2	42.3	72.0
Nurses	2011	09.0	3.0	11.5	3.1	35.5	44.3	70.4
General population	2014-2015	13.3	6.1	18.8	9.0	44.5	72.0	74.6

Kontis et al. studied morbidity trends and NCDs risk factors in different regions of the world concluding that only the United States and Europe will achieve 25×25 target. Other countries were likely to reach the target in case they could reduce the risk factors to the specified levels ^1^. In their cohort study of the Swedish population, Santosa et al. tried to find out whether they could achieve the target. They asserted that countries such as Sweden, which have been on an epidemiological transition in 2010, have already managed to reach the desired 25% reduction. They consider it as a piece of good news for other countries, suggesting that other countries can also obtain 25% reduction of morbidity from NCDs if they take proper measures ^4^.

In Lancet Journal, Helen Clark writes that such a target cannot be achieved unless there is collaborative action from health sectors led by WHO ^5^. NCD prevention and control, she adds, can be worked both in practice and in policy. In terms of practice, development stakeholders can contribute to the prevention and control of NCBs in their routine settings such as workplaces, schools, and other public sector institutions. In terms of policy, a number of regulations including taxation, production, advertising restrictions, or regulation of consumption can positively affect NCD prevention and control. Nonetheless, the challenge concerns how to maximize chances for positive cooperation between sectors.

Given the progressive trend of cardiovascular risk factors in eastern and other regions of Iran^6^, therefore, a public mobilization is required to prevent NCDs. The government should give it a priority in the health system, and all organizations should cooperate with the Ministry of Health, Care, and Medical Education if NCBs are going to be overcome.

## Acknowledgements

The authors declare that there is no conflict of interests.

**Masoud Siadat (MD)** ^a,b^ **, Toba Kazemi (MD)** ^a*^ **, Morteza Hajihosseni (MSc)** ^a^

^a^ *Atherosclerosis and Coronary Artery Research Center, Birjand University of Medical Sciences, Birjand, Iran*

^b^ *South Khorasan Health Insurance, Birjand, Iran*

*****Correspondence to:***** *Toba Kazemi (MD)*

***E-mail:*** drtooba.kazemi@gmail.com
